# Pulsed Ultraviolet Light Reduces Immunoglobulin E Binding to Atlantic White Shrimp (*Litopenaeus setiferus*) Extract

**DOI:** 10.3390/ijerph8072569

**Published:** 2011-06-24

**Authors:** Sandra Shriver, Wade Yang, Si-Yin Chung, Susan Percival

**Affiliations:** 1 Department of Food Science & Human Nutrition, University of Florida, P.O. Box 110370, 359 FSHN Bldg. Newell Drive, Gainesville, FL 32611, USA; E-Mails: sandrakshriver@ufl.edu (S.S.); percival@ufl.edu (S.P.); 2 Southern Regional Research Center, Agricultural Research Service, U.S. Department of Agriculture, 1100 Robert E. Lee Blvd., Bldg 001 SRRC, New Orleans, LA 70124, USA; E-Mail: siyin.chung@ars.usda.gov

**Keywords:** allergen, allergy, shrimp, pulsed ultraviolet light, PUV, tropomyosin, IgE antibodies

## Abstract

Pulsed ultraviolet light (PUV), a novel food processing and preservation technology, has been shown to reduce allergen levels in peanut and soybean samples. In this study, the efficacy of using PUV to reduce the reactivity of the major shrimp allergen, tropomyosin (36-kDa), and to attenuate immunoglobulin E (IgE) binding to shrimp extract was examined. Atlantic white shrimp (*Litopenaeus setiferus*) extract was treated with PUV (3 pulses/s, 10 cm from light source) for 4 min. Tropomyosin was compared in the untreated, boiled, PUV-treated and [boiled+PUV]-treated samples, and changes in the tropomyosin levels were determined by sodium dodecyl sulfate-polyacrylamide gel electrophoresis (SDS-PAGE). IgE binding of the treated extract was analyzed via immunoblot and enzyme-linked immunosorbent assay (ELISA) using pooled human plasma containing IgE antibodies against shrimp allergens. Results showed that levels of tropomyosin and IgE binding were reduced following PUV treatment. However, boiling increased IgE binding, while PUV treatment could offset the increased allergen reactivity caused by boiling. In conclusion, PUV treatment reduced the reactivity of the major shrimp allergen, tropomyosin, and decreased the IgE binding capacity of the shrimp extract.

## 1. Introduction

In the United States, approximately 6% of children and 3.7% of adults are affected by one or more food allergies. Eight major food sources, including cow’s milk, shellfish, egg, fish, tree nuts, peanuts, soybean, and wheat, account for approximately 85% of all food allergies [1]. Allergies to shellfish, such as shrimp, affect 0.1% of American children and 2% of American adults, making shellfish allergies the most common type of food allergy in adults [2].

The major heat-stable allergen of shrimp is a 36-kDa protein known as tropomyosin, also referred to as Sa-II or Pen a 1 [3–5]. Tropomyosin plays an important role in muscular contraction, as well as in the regulation of cellular structure and motility [6]. Although present in both vertebrates and invertebrates, tropomyosin is known to elicit an allergic reaction only when it is derived from invertebrate sources, such as crustaceans, arachnids, insects and mollusks [5]. Two additional shrimp allergens have been identified: myosin light chain (20-kDa) [7] and arginine kinase (40-kDa) [8–10]. However, studies suggest that the majority of shrimp allergenicity can be attributed to tropomyosin protein alone [3,5,11], where tropomyosin was recognized by 82% of patients with shrimp allergies and was shown to inhibit IgE binding to whole-body shrimp extract in 85–95% of patients.

According to Jeong and others [6], the most frequent symptoms of shrimp-induced allergies include itching, hives, swelling of the lips and tongue, pulmonary symptoms, gastrointestinal symptoms, and anaphylactic shock. Methods such as oral and sublingual immunotherapy have been used in attempt to prevent shrimp-induced allergies, yet the only completely effective approach to date is total avoidance [12]. Complete avoidance is often difficult and inconvenient, considering the prevalence of food allergens in a multitude of products. Thus, researchers are seeking diverse ways, such as postharvest treatment methods, to reduce the allergen reactivity of food products before they reach the consumer. When an allergen enters the body, immunoglobulin E (IgE) antibodies elicit an immune response by binding to specific epitopes on an allergen, which can be linear or conformational [13]. Postharvest methods have the potential to alter these epitopes by disrupting or masking amino acid sequences (e.g., protein fragmentation or genetic modification) or by altering the conformation of the protein (e.g., protein denaturation, protein crosslinking, or aggregation). Postharvest methods, including power ultrasound [14], gamma irradiation [15], and high hydrostatic pressure processing [16], have been shown to alter allergen reactivity by modifying allergen structure. More recently, pulsed ultraviolet light (PUV), an emerging technology, has been employed to reduce the allergen reactivity of peanut products [17,18] and soy extracts [19].

Pulsed ultraviolet light is considered more effective in food processing (specifically microbial inactivation) than conventional, or continuous, UV light, because of its instantaneous high-energy pulses and greater capability to penetrate. In a PUV system, electrical energy is captured and stored in a capacitor and is ultimately released in short pulses as ultraviolet, infrared, and visible light (approximately 54%, 20%, and 26%, respectively) [20]. The resultant bursts can be several thousand times more intense than continuous UV light [21]. Upon coming into contact with a sample, the light interacts with molecules, which are excited and–upon returning to ground state–liberate energy as photons or heat, which can induce chemical changes. Thus the efficacy of PUV has been attributed to photochemical, photothermal, and photophysical reactions [21]. These effects may contribute to changes in protein structure and reduction in IgE binding to allergens.

At a shorter exposure (e.g., seconds), PUV is normally regarded as nonthermal, as the temperature rise of food is insignificant. However, at a longer exposure (e.g., minutes), PUV can generate significant photothermal effects and incur considerable temperature rise and moisture loss to the sample [17–19,22–25]. It has also been found that prolonged UV light treatment caused formation of insoluble complexes in food, depolymerization of starch, peroxidation of unsaturated fatty acids, carbohydrate crosslinking, protein crosslinking, and protein fragmentation [26–29]. The significant photothermal effect of PUV after an extended exposure enables the PUV technology to be applied directly to solid foods, e.g., whole almond [23], for allergen mitigation. Li [23] exposed the whole almond kernels to PUV for 4–7 min. It was found the whole almond treated with PUV for 4 min had a desirable and pleasurable roasted almond flavor and taste, and its IgE biding capacity was pronouncedly reduced, although the exact mechanism of PUV interactons with the solids is still unknown.

Based on the studies of PUV treatment on peanut and soybean allergens [17–19], we hypothesized that PUV treatment of Atlantic white shrimp extract would alter the reactivity of the major shrimp allergen, tropomyosin, and consequently reduce the overall allergenic potential of the shrimp extract. Our objective was to examine the efficacy of PUV exposure on the inactivation of major shrimp allergen by measuring changes in tropomyosin level and IgE binding.

## 2. Experimental Section

### 2.1. Materials

Frozen Atlantic white shrimp (*Litopenaeus setiferus*) were purchased de-headed and shelled from Publix Supermarkets, Inc. (Lakeland, FL). Coomassie Plus (Bradford) Protein assay, bovine serum albumin (BSA), StartingBlock/Tris-buffered saline/Tween-20 (StartingBlock), GelCode Blue gel staining reagent, *o*-phenylenediamine dihydrochloride (OPD) and SuperSignal West Pico Chemiluminescent (ECL) substrate were purchased from Thermo Fisher Scientific Inc. (Rockford, IL). Electrophoresis equipment and reagents including pre-cast Tris-HCL minigels (4–15%), Mini-PROTEAN^®^ Tetra cell tanks, Laemmli sample buffer, Tris-glycine transfer buffer, Tris-glycine-SDS running buffer, nitrocellulose membrane (0.45 μm) and Trans-blot SD Semi-dry Transfer cells were purchased from Bio-Rad Laboratories, Inc. (Hercules, CA). Pooled human plasma from 3 patients with history of shrimp allergy was obtained from PlasmaLabs International (Everett, WA) for immunoblotting and enzyme-linked immunosorbent assay (ELISA). The plasma-specific IgE level for pooled human plasma with antibodies specific to shrimp was measured by the ImmunoCAP method performed by PlasmaLabs and was determined to be 92 kU L^−1^. A secondary-detection antibody, mouse anti-human IgE conjugated to horseradish peroxidase (HRP), was obtained from Invitrogen (Carlsbad, CA). Rat monoclonal anti-tropomyosin (IgG isotype) and rabbit polyclonal anti-rat IgG-H&L conjugated to HRP antibodies were purchased from Abcam Inc. (Cambridge, MA). Costar Enzyme Immunoassay (EIA) polystyrene 96-well plates (Corning, NY) and Immobilon P blotting polyvinylidene fluoride (PVDF) membrane (0.45 μm) (Millipore Corporation, Bedford, MA) were used for immunological assays.

### 2.2. Preparation of Atlantic White Shrimp Crude Protein Extract

Shrimp extract was prepared following the methods of Motoyama and others [30,31] with modification. Briefly, shrimp (25 g) was ground in a food processor at low speed for 10 s. A volume of 0.6 M KCl in 0.01 M phosphate buffer (200 mL, pH 7) was added to the processed tissue, and the mixture was homogenized at the high-speed setting (10,000 rpm) for 1 min using a BioSpec BioHomogenizer (Bartlesville, OK). The buffer was carefully selected in order to solubilize the major allergen while maintaining its native state. Protein concentration was measured with Bradford assay using BSA protein standards. The extract was diluted to a concentration of 5 mg/mL with 0.6 M KCl in 0.01 M phosphate buffer (pH 7). Samples were treated immediately or stored packaged on ice in a styrofoam container placed at 4 °C for no longer than one week.

### 2.3. Preparation of Boiled Shrimp Extract

A common way of preparing shrimp is by boiling, but literature shows the allergen immunoactivity of shrimp is not reduced in general or in some cases is even increased by boiling [32–34]. Consumption of boiled or steamed shrimp resulted in more severe allergy skin test responses in some patients than raw shrimp [33,34]. Liu and others [35] reported that although iELISA demonstrated that the raw shrimp extracts had higher IgE binding than the boiled shrimp extracts, dot-blot results showed higher IgE binding to the purified tropmyosin from boiled shrimp than raw shrimp. In this study, boiled shrimp extracts were also prepared by heating in boiling water for 4 min and then tested for its IgE binding capacity, which was compared to the PUV treated samples. A volume of 10 mL of crude shrimp protein extract was placed in boiling water for 4 min in a loosely capped 15 mL centrifuge tube. Following heat treatment, samples were cooled on ice, and protein concentration was measured.

### 2.4. Treatment of Shrimp Extract with PUV

Raw and boiled samples (10 mL, 5 mg/mL) were treated with a Xenon Steripulse-XL RS-3000 batch PUV sterilization unit (Wilmington, MA), as described by Chung and others [17], to examine the effect of PUV on the immunoreactivity of both raw and boiled shrimp samples. The treatment of boiling followed by PUV was to exemine if synergistic or antagonistic effect on allergen reactivity existed between boiling and PUV treatments.

Pulses were emitted at a rate of 3/s with a pulse width of 360 μs, and samples were positioned at a distance of 10 cm from the quartz window of the PUV lamp in aluminum dishes with a diameter of 7.2 cm. Under these conditions, the maximum energy level of the emitted radiation was 0.27 J/cm^2^ per pulse as per the factory calibration. Temperature of the samples was recorded using an Omega OS423-LS non-contact infrared thermometer (Omega Engineering, Inc., Stamford, CT), and the samples were cooled on ice following treatment. Changes in volume were measured, and protein concentration was determined with Bradford assay.

### 2.5. Electrophoresis of Treated Shrimp Extract

Samples were analyzed under reducing conditions as described by Laemmli [36]. Briefly, a sample containing protein (12 Rg) was combined with sample buffer (62.5 mM Tris-HCL, pH 6.8, 2% SDS, 25% glycerol, 0.01% bromophenol blue, 0.05% β-mercaptoethanol) in a microcentrifuge tube. The mixture was heated in boiling water for 5 min. The sample was then subjected to electrophoresis within a 4–15% Tris-glycine gel for 1.5 h at 150 V per the manufacturer’s recommendations. Subsequently, the gel was stained with GelCode Blue reagent for 2 h and destained for 1 h with deionized water. The protein bands were scanned with a Canon Pixma MP160 scanner.

### 2.6. Determination of IgE- and IgG-Binding to Tropomyosin with Western Blot

Following electrophoresis, proteins were transferred onto a PVDF blotting membrane at 15 V for 30 min. Nonspecific binding sites were blocked for 1 h at room temperature (RT) with StartingBlock blocking buffer. The membrane was incubated overnight at 4 °C with pooled human plasma containing anti-shrimp IgE antibodies (1:80) diluted in blocking buffer. After washing with Tris buffered saline containing 0.1% Tween 20 (TBST), the blot was then incubated in mouse anti-human IgE-HRP diluted 1:1,000 in blocking buffer for 1 h at RT. The blot was again washed, and incubated in SuperSignal West Pico Chemiluminescent substrate for 5 min at RT. The protein bands were developed on an X-ray film. To visualize changes in tropomyosin band intensity, the above procedure was replicated, replacing the primary antibody with rat monoclonal anti-tropomyosin (IgG) (1:1,000) and the secondary antibody with rabbit polyclonal anti-rat IgG-HRP (1:40,000).

### 2.7. Determination of IgE Binding to Treated Shrimp Extract with Dot Blot

A nitrocellulose membrane was blotted with 1.25 and 2.5 μg of raw, boiled, PUV-treated, and [boiled+PUV]-treated shrimp extract protein and allowed to dry at 4 °C. Following blocking with StartingBlock blocking buffer, the blot was incubated overnight at 4 °C in a pooled human plasma containing anti-shrimp IgE antibodies (1:80) diluted in blocking buffer. The blot was washed and then incubated in mouse anti-human IgE-HRP diluted 1:1,000 in blocking buffer (1 h; RT). The blot was again washed, and incubated in SuperSignal West Pico Chemiluminescent substrate for 5 min at RT. The protein spots were developed on an x-ray film.

### 2.8. Determination of IgE Binding to Treated Shrimp Extracts with Indirect ELISA

Polystyrene 96-well plates were coated overnight at 4 °C with untreated, boiled, PUV-treated, and [boiled+PUV]-treated shrimp extract diluted in phosphate-buffered saline (PBS) to a concentration of 20 μg/mL (100 μL per well in triplicates). The plates were subsequently washed with TBST and blocked with StartingBlock blocking buffer (200 μL per well) at room temperature for 2–3 h. Pooled human plasma containing IgE antibodies specific for shrimp allergens was diluted in PBS (1:10) and added in equal amounts to each well (100 μL). The plate was incubated at room temperature with gentle shaking for 1 h. After washing with TBST, each well was then incubated with secondary antibody, monoclonal mouse anti-human IgE conjugated to HRP (1:3,000), for 1 h (100 μL per well) with gentle shaking. The wells were again washed, and an OPD substrate (0.5 mg/mL) dissolved in 0.1 M citrate buffer (pH 5.5) and 0.03% hydrogen peroxide was added to each well (100 μL per well). The reaction was stopped at 15–30 min with 2.5 N sulfuric acid (100 μL per well), and absorbance was measured at 490 nm using a Spectramax 340^384^ spectrophotometer (Molecular Devices, Inc. Sunnyvale, CA).

### 2.9. Statistical Analysis

Statistical analysis was conducted using one-way analysis of variance (ANOVA) with the SAS 9.2 software package (Cary, N.C.). Significant differences (S = 0.05) between means of the untreated (control) and treated (boiled, PUV-treated, and [boiled+PUV]-treated) samples for total IgE binding were determined using least significant difference (LSD) and Duncan’s Multiple Range tests.

## 3. Results and Discussion

### 3.1. Optimal PUV Treatment Time for Shrimp Extract

**Figure 1:**
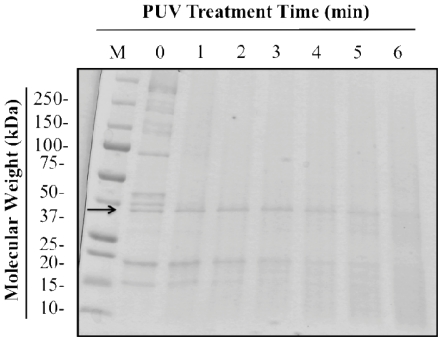
An SDS-PAGE profile of shrimp extract treated with PUV at 0, 1, 2, 3, 4, 5, and 6 min. Molecular weight marker (M) is shown. The band corresponding to tropomyosin (36-kDa) is highlighted with an arrow.

To establish an appropriate treatment time, several time courses for PUV treatment were tested. SDS-PAGE and Western blot analyses were used to determine the minimum exposure time at which PUV treatment led to a reduction in both tropomyosin level and IgE binding. An SDS-PAGE analysis (Figure 1) illustrated that tropomyosin bands (36-kDa) remained in the extract following PUV treatments of 1, 2, and 3 min, but were reduced in samples treated for 4–6 min. Densitometry analysis verified the reduction in tropomyosin band intensity at 4–6 min. It is noted that Figure 1 also shows other two minor allergenic proteins (16.5-kDa and 20-kDa), which have also been reported in other studies [37].

**Figure 2:**
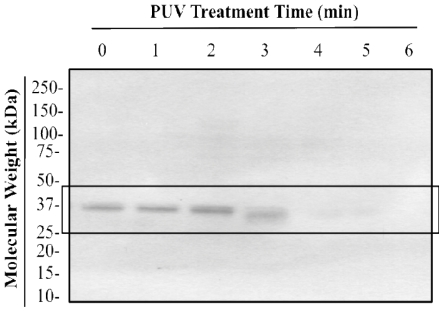
Western blot analysis of shrimp extract samples treated with PUV at 0 (control), 1, 2, 3, 4, 5, and 6 min using pooled human plasma from 3 individuals containing IgE antibodies against shrimp. Tropomyosin bands (36-kDa) are highlighted within a box.

Western blot analysis of the PUV-treated extracts at 0–6 min (Figure 2) showed that IgE binding to tropomyosin was markedly reduced at 4–6 min, compared to 0–3 min. Because patterns between 4 and 6 min were similar and a higher moisture loss was observed at 6 min, 4 min was chosen as the optimal treatment time for subsequent experimentation.

### 3.2. Changes in Tropomyosin Band Intensity of Untreated, Boiled, PUV-Treated, and [Boiled+PUV]-Treated Shrimp Extracts

**Figure 3:**
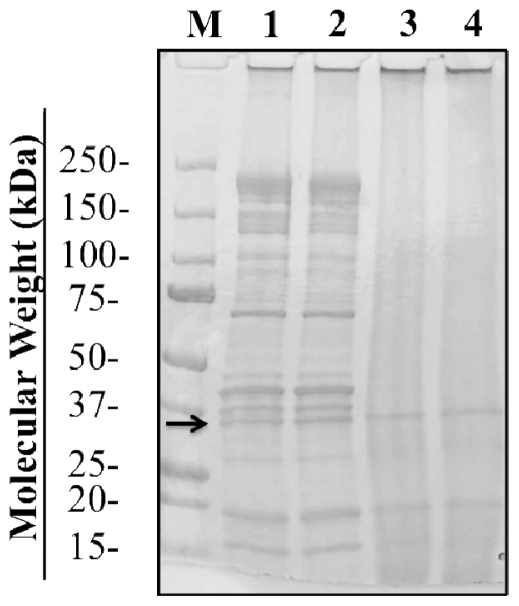
An SDS-PAGE profile of untreated (1), boiled (2), PUV-treated (3), and [boiled+PUV]-treated (4) shrimp extracts. Molecular weight marker is shown (M). An arrow highlights the bands corresponding to tropomyosin (36-kDa).

As illustrated in the SDS-PAGE profile (Figure 3), a 36-kDa band representing tropomyosin was present following treatments with boiling, PUV, and boiling+PUV. A decrease in tropomyosin was observed in both PUV- and [boiled+PUV]-treated samples (lanes 3 and 4), whereas the boiled extract (lane 2) did not show a change in tropomyosin density compared to the control. These results were verified using densitometry analysis.

Like Figure 1, Figure 3 also shows the behavior of other two allergenic proteins (16.5-kDa and 20-kDa) under boiling and PUV treatments. The 20-kDa protein followed a similar trend to tropomyosin’s in terms of boiling and PUV effects, while boiling alone did not change much the 16.5-kDa protein, but PUV caused it to be almost undetectable as illustrated on lanes 3 and 4.

Resistance to thermal denaturation or degradation is characteristic of the tropomyosin protein, as it is recognized for its heat stability [3]. However, instantaneous pulses of energy generated during prolonged PUV treatment may cause more intense localized heating [38,39], which may contribute to the reduction of tropomyosin. On the other hand, UV exposure, which is basically nonthermal, can also cause protein crosslinking or fragmentation [27,29]. So, we believe both the nonthermal and photothermal effects of PUV played a role in reducing the allergenic reactivity of shrimp proteins in this study.

### 3.3. IgE- and IgG-binding to Untreated, Boiled, PUV-treated, and [Boiled+PUV]-Treated Shrimp Extracts

#### 3.3.1. Western Blot

**Figure 4:**
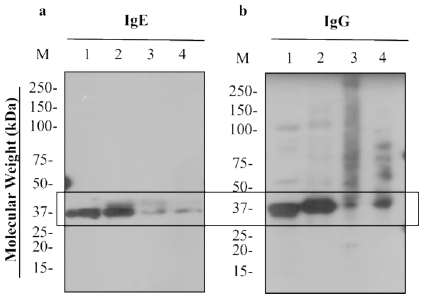
Western blots using (**a**) pooled human plasma containing IgE antibodies against shrimp and (**b**) monoclonal anti-tropomyosin antibody (IgG) to analyze untreated (1), boiled (2), PUV-treated (3) and [boiled+PUV]-treated (4) shrimp extracts. Tropomyosin (36-kDa) is highlighted using a box.

The four treated samples were analyzed with Western blot using IgE antibodies against shrimp, to evaluate allergen reactivity, and an anti-tropomyosin antibody (IgG), to evaluate tropomyosin levels. Figure 4 demonstrates a notable decrease in IgE binding and tropomyosin levels following PUV treatment, as evidenced by decreases in band intensity. The marked difference between the control and PUV treatment further supports the SDS-PAGE data (Figure 1) that tropomyosin levels were indeed reduced at 4 min. Boiling, however, appeared to increase IgE binding, based on densitometry analysis of the control versus boiled.

Presently, it is still unknown exactly how the IgE binding was reduced by PUV radiation, but it is believed to relate to localized photochemical, photothermal and photophysical effects of PUV, which, as mentioned earlier, can cause protein modifications, including protein fragmentation, denaturation, or crosslinking and affect IgE binding. Fragmented protein sections that are smaller than the resolution limit of the gel may travel through the acrylamide pores more quickly, causing them to ultimately be lost into the chamber buffer. Conversely, proteins that have been modified by crosslinking are often too large to migrate through the gel or migrate with slight difficulty, thereby resulting in a smeared appearance in SDS-PAGE and Western blots. Chung and others [40] have demonstrated the presence of protein smears due to the crosslinking of peanut allergens caused by enzymatic reactions. Also, protein band smearing has been observed in the allergens of peanuts that have been heated or roasted [41]. While band smearing was not detected in the blot with IgE (Figure 4a), they can be seen in blots using an anti-tropomyosin antibody. As shown in Figure 4b, tropomyosin bands and others were notably smeared in PUV-treated samples, but not in boiled and control samples. This finding indicates that modification or crosslinking of tropomyosin might have occurred during the PUV treatment.

Additionally, Taheri-Kafrani and others [42] have illustrated the smearing of milk allergens that have been modified via the Maillard reaction, which is a non-enzymatic browning or protein-carbohydrate reaction. Several studies [42–44] have linked glycation or Maillard reaction to the modification of allergens and their subsequent changes in IgE binding. A study on squid tropomyosin [44] found that IgE binding was suppressed with the progression of the Maillard reaction. However, in one study using scallop tropomyosin [43], an increase in the allergen potency was displayed with the progression of Maillard reaction. Whether Maillard reaction could occur in the shrimp extract during PUV treatment is not known. Considering the high energy produced by PUV and the instantaneous heat absorbed by the molecules [39] (*i.e.*, proteins/carbohydrate in the shrimp extracts), it is possible that a Maillard reaction may occur, but such a postulation needs to be further investigated.

#### 3.3.2. Dot Blot

**Figure 5:**
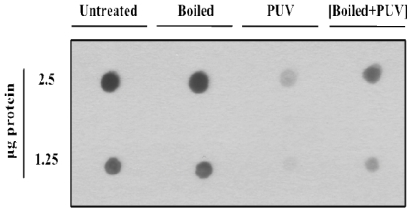
Dot blot analysis of untreated, boiled, PUV-treated, and [boiled+PUV]-treated shrimp extract using pooled human plasma containing IgE antibodies against shrimp.

Dot blot analysis was performed to determine the allergen reactivity of the shrimp protein extract as a whole–that is, tropomyosin and other possible allergens that were not detected via Western blot analysis. IgE binding to whole shrimp extract was greatly reduced following PUV treatment (Figure 5). However, there was an increase in IgE binding following boiling treatment. Interestingly, PUV appeared to attenuate or negate the boiling effect, as shown in the [boiled+PUV]-treated sample; IgE binding was notably reduced compared to the boiled-only sample. Of all the treatments, PUV alone displayed the most reduction in IgE binding.

The finding that boiling or heating can lead to an increase in IgE binding (Figure 5) is not unusual, because the effects of thermal processing on food allergens have been studied extensively, and, in different studies, heating has been shown to either decrease or increase allergen potency. For example, one study [41] described a 90-fold increase in IgE binding of roasted peanuts over raw peanuts, whereas another study reported that roasting actually decreased the overall allergen reactivity of hazelnuts [45]. It has also been noted that children with milk allergies show a tolerance to extensively heated milk [46]. In this dot blot analysis (Figure 5), boiling caused an increase in IgE binding possibly not just due to tropomyosin itself, but also due to other proteins present in the whole extract. These proteins could include arginine kinase (40-kDa) [8,9] and myosin light chain (20-kDa) [7,38] which have both been shown to play a minor role in shrimp allergy. Also, the minor 16.5 kDa protein was also persistent during the boiling treatment (Figure 3). The effects of boiling on shrimp reactivity are consistent with the results of Carnes and others [32] who also found that boiled shrimp extracts were more immunoreactive in both *in vivo* skin prick trials and *in vitro* direct ELISA results.

#### 3.3.3. Indirect ELISA

**Figure 6:**
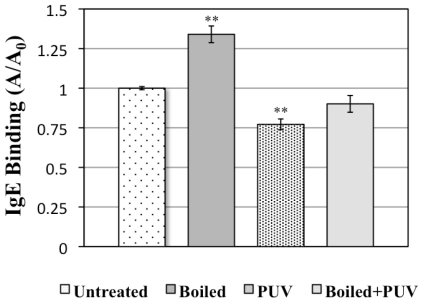
Indirect ELISA illustrating changes in IgE binding compared to untreated, boiled, PUV-treated, and [boiled+PUV]-treated shrimp extracts using pooled human plasma containing IgE antibodies against shrimp. A = absorbance of the sample; A_0_ = absorbance of untreated sample. Data are expressed as mean ±SEM (n = 5). Results are relative values, normalized to the untreated sample; untreated is standardized and set to 1. Values that are significantly different (S = 0.05) from the untreated sample are annotated as **.

To support the dot blot data in Figure 4, IgE binding of the four treated shrimp extracts was also determined using an indirect ELISA. Again, a significant decrease (S = 0.05) in IgE binding was seen in the PUV-treated extract, compared to the control (Figure 6). An increase in IgE binding was observed in the boiled extract while there was no significant change (S = 0.05) in IgE binding of the [boiled+PUV]-treated extract, as compared to the control. The finding was in agreement with the data of dot blot (Figure 4).

Reduction in IgE binding of PUV-treated extract was likely explained by changes in the amount of detectable tropomyosin described above in SDS-PAGE (Figures 1 and 3) and Western blot analysis (Figures 2 and 4). In the [boiled+PUV]-treated sample, IgE binding did not appear to change, compared to the control. This was because tropomyosin increased after boilng but was offset to the control level after the PUV treatment. That is to say, the increase and decrease in IgE binding, respectively, due to boiling and PUV treatment, may negate each other, thus resulting in a negligible change in IgE binding of the [boiled+PUV] extract. Such an effect may be deemed as an antagonistic effect.

### 3.4. Temperature and Volume Changes Following Treatment of Shrimp Extract

As mentioned earlier, PUV is considered a nonthermal method when used for brief periods of time (several seconds); however, following a PUV treatment period of 4 min, a sample surface temperature of 68.3 ± 2.5 °C was detected using an infrared thermometer immediately after the PUV pulses stopped and the treatment chamber door was opened. It must be noted that there was a 5–10 s delay in probing the sample surface temperature, while the instantaneous temperatures of the sample during the PUV treatment could be higher. Previous trials using thermocouples to monitor temperature during PUV treatment were unsuccessful, because extended PUV exposure of the metal probe confounded the readings. Fiber optic temperature sensing may be a way to go for recording accurately the sample temperature during PUV treatment, which was not conducted in this study. For shorter PUV treatments, a type-K thermocouple produced by Omega Engineering, Inc. (Stanford, CT), has been utilized [47] for temperature measurement without confounding a complication in temperature reading. A study that analyzed PUV treatment for decontamination of shell-eggs [47] reported an increased surface temperature of 10.5 ± 1.2 °C after 30 s at 9.5 cm distance from the quartz window of the PUV lamp.

Following boiling, PUV, and [boiling+PUV] treatments, moisture loss in each sample was measured: boiled-only (5.83 ± 2.3%), PUV-only (29 ± 3.6%), and boiled with PUV (39.7 ± 2.5%). Moisture loss was higher in the PUV-treated samples because the samples were not enclosed during PUV treatment (the purpose was to ensure the maximum absorption of the PUV radiation). By contrast, moisture loss was minimal in the boiled sample, because the samples were loosely capped during boiling. Chung and others [17] also noted volume reductions of approximately 40% following PUV treatment. To correct for moisture loss in the samples, protein measurements were taken after treatments, and these values were used for subsequent experiments.

Excessive moisture loss is indicative of a sample temperature during the PUV treatment that was well above the boiling point of water at atmosphere, which caused the water to evaporate. The significant temperature increases due to the photothermal effect of PUV and the capability of PUV in mitigating allergens may potentially be used in conjunction with food preparation to simultaneously heat the food and reduce its allergenic potency. Li [23] took advantage of this unique feature of PUV radiation to desirably roast the whole almond after 4–7 min exposure and yet considerably reduce its IgE biding capacity. This can also be a potential method to cook the peeled whole shrimp and significantly reduce its allergen, although this idea was not tested yet in this study.

## 4. Conclusions

A marked decrease in IgE binding of the shrimp extract following PUV treatment has been demonstrated. The decrease was likely due to a reduction in the detectable level of tropomyosin in the PUV-treated extract as shown in SDS-PAGE (Figure 1) and Western blot (Figure 2). Furthermore, the appearance of protein band smearing, as illustrated in Figure 4, is likely due to the modification, such as crosslinking, of tropomyosin and may contribute to the decrease in IgE binding. Boiling increased IgE binding to whole shrimp sample; however, the effect of boiling was offset when it was combined with PUV treatment. Overall, PUV was found to be capable of reducing the allergenic potency of shrimp extracts. Further optimization is still needed before the PUV technology can be adopted. In vivo studies are also needed to verify the reduction in allergenic potency of the PUV-treated shrimp extracts.
